# Joint Use of Genome, Pedigree, and Their Interaction with Environment for Predicting the Performance of Wheat Lines in New Environments

**DOI:** 10.1534/g3.119.400508

**Published:** 2019-07-12

**Authors:** Réka Howard, Daniel Gianola, Osval Montesinos-López, Philomin Juliana, Ravi Singh, Jesse Poland, Sandesh Shrestha, Paulino Pérez-Rodríguez, José Crossa, Diego Jarquín

**Affiliations:** * University of Nebraska – Lincoln, Lincoln NE, 68583, USA; † University of Wisconsin-Madison, Madison, Wisconsin 53706, USA; ‡ Universidad de Colima, CP 28040 Colima, México; § International Maize and Wheat Improvement Center (CIMMYT), Km. 45, Carretera México-Veracruz, El Batán, Texcoco, Edo. de México, CP 56130, México; ** USDA-ARS and Dep. of Plant Pathology, Kansas State University, 4024 Throckmorton Hall, Manhattan KS, 66506, USA, and; †† Colegio de Postgraduados, Montecillos, Edo. de México, CP 56230, México

**Keywords:** genome-enabled prediction, pedigree-enabled prediction, genomic × environment interaction, pedigree × environment interaction, genomic × pedigree × environment interaction, CIMMYT wheat evaluation trials, Genomic Prediction, GenPred, Shared Data Resources

## Abstract

Genome-enabled prediction plays an essential role in wheat breeding because it has the potential to increase the rate of genetic gain relative to traditional phenotypic and pedigree-based selection. Since the performance of wheat lines is highly influenced by environmental stimuli, it is important to accurately model the environment and its interaction with genetic factors in prediction models. Arguably, multi-environmental best linear unbiased prediction (BLUP) may deliver better prediction performance than single-environment genomic BLUP. We evaluated pedigree and genome-based prediction using 35,403 wheat lines from the Global Wheat Breeding Program of the International Maize and Wheat Improvement Center (CIMMYT). We implemented eight statistical models that included genome-wide molecular marker and pedigree information as prediction inputs in two different validation schemes. All models included main effects, but some considered interactions between the different types of pedigree and genomic covariates via Hadamard products of similarity kernels. Pedigree models always gave better prediction of new lines in observed environments than genome-based models when only main effects were fitted. However, for all traits, the highest predictive abilities were obtained when interactions between pedigree, genomes, and environments were included. When new lines were predicted in unobserved environments, in almost all trait/year combinations, the marker main-effects model was the best. These results provide strong evidence that the different sources of genetic information (molecular markers and pedigree) are not equally useful at different stages of the breeding pipelines, and can be employed differentially to improve the design and prediction of the outcome of future breeding programs.

It is important to increase food production to meet the needs of an increasing world population (Whitford *et al.*, 2013), and satisfy people’s dietary requirements. Wheat is a major cereal grain worldwide and the third most-produced cereal after maize and rice. It is an important source of protein and calories and the dominant staple in North Africa, West and Central Asia, and the European Union (Tadesse *et al.*, 2017). The Consultative Group for International Agricultural Research (CGIAR) estimated that, by 2050, wheat production would need to increase by 60% to satisfy consumers’ needs. Such an objective cannot be met with contemporary techniques because wheat production is currently increasing by less than 1% per year (Ray *et al.*, 2013). Thus, there is an urgent need to develop methods that would make it possible to increase wheat production to meet the current and future global demand.

In plant and animal breeding, predicting the breeding values of individuals depends, among other factors, on the target population, the genetic complexity of the traits, and the target environments where the traits are to be measured. In particular, efficient plant breeding programs need to address genotype × environment interactions (G×E) that reflect differential reactions of certain genotypes across a set of environmental circumstances. Environmental conditions affecting gene expression induce G×E, and estimates of genetic correlations of line performance across environments summarize the collective action of genes and environmental conditions. In theory, the correlation between performances in different environments may vary across chromosome regions, and standard mixed models for assessing G×E typically do not decompose the total G×E into specific chromosome region × environment interactions.

Bernardo (1994) was the first to propose using markers in pedigree-related lines for predicting yet-to-be-observed individual crosses. Subsequently, Meuwissen *et al.* (2001) proposed whole genome regression models for genomic selection (GS) where hundreds of thousands of markers were fitted jointly as covariates for predictive purposes. When the number of markers (*p*) is larger than the number of individuals (*n*), regularization methods are often beneficial for prediction. Proper prior distributions in Bayesian models produce regularization, but can have a marked effect on inference when *n*<<*p* (Gianola *et al.*, 2009; de los Campos *et al.*, 2013; Gianola, 2013). Empirical evidence obtained with plant and animal breeding data has shown that GS often outperforms pedigree-based prediction methods by a sizable amount (de los Campos *et al.*, 2009; Crossa *et al.*, 2010). GS has become a useful approach for improving quantitative traits of many crops in plant breeding.

Wheat production and its profitability are highly influenced by environmental factors, especially climate. The interaction between environmental factors and genotypes also affects wheat yield. Hence, environmental factors and G×E interaction must be included in prediction models. The first study using a model including genome × environment interaction was conducted by Burgueño *et al.* (2012). These authors developed GS models for multi-environmental trials based on the genomic best linear unbiased predictor (GBLUP), using the notion of genetic correlations between environments (Falconer, 1952). Burgueño *et al.* (2012) found that the multi-environmental GBLUP had higher prediction accuracy than the single-environment GBLUP. Later, Jarquín *et al.* (2014) proposed random effects models where main effects and interaction effects between markers and environmental covariates were introduced using covariance structures as in reproducing kernel Hilbert spaces (RKHS) regression (*e.g.*, Gianola *et al.*, 2006; Gianola and van Kaam, 2008). The approach presented by Jarquín *et al.* (2014) is an extension of GBLUP, and can also be interpreted as a reaction norm model where genetic and environmental gradients are represented by linear functions of markers and environmental covariates (*e.g.*, Gregorius and Namkoong, 1986; Falconer and Mackay, 1996; Calus *et al.*, 2002; Calus and Veerkamp, 2003). The advantage of the GBLUP model of Jarquín *et al.* (2014) is that it allows using not only large numbers of markers and environmental covariates, but also high-dimensional additive relationship matrices obtained from the pedigree information.

The model of Jarquín *et al.* (2014) has been used successfully for multi-environmental data and for incorporating interactions among multi-type input sources (*e.g.*, dense molecular markers, pedigree, high-throughput phenotypes) in several crops (Pérez-Rodríguez *et al.*, 2015; Crossa *et al.*, 2016; Sousa *et al.*, 2017; Crain *et al.*, 2018; Jarquín *et al.,* 2018). Many studies using the Jarquín *et al.* (2014) model indicated that including G×E interaction in the model substantially increased the accuracy of across-environment (locations and/or years) predictions (Crossa *et al.*, 2017).

Most studies involving pedigree or genome × environment interaction do not include more than 1000 lines per year in different environments (year by location combinations), with all lines repeated in all environments (Crossa *et al.*, 2017). Pérez-Rodríguez *et al.* (2017) was the first study in which GBLUP and pedigree models were used to assess the prediction accuracy of a large number of CIMMYT wheat lines (58,798) evaluated in several site-year combinations in México, and to predict grain yield performance of wheat lines in several sites of South Asia using the GBLUP with G×E model. The predictive correlation of models using only pedigree, only markers, or both pedigree and markers to predict performance in South Asia (India, Pakistan, and Bangladesh) was around 0.25-0.38, which was higher than the correlation attained using phenotypic values only (0.20).

To our knowledge, no studies have addressed the potential interaction between pedigree and genomic information, and between pedigree, genomic, and environmental information for a large number of lines evaluated for several years where lines are tested for only one year. If genome-enabled prediction is more effective when individuals are genetically (pedigree) related than when they are nominally unrelated (*e.g.*, Habier *et al.*, 2007), the interaction between pedigree and markers and between marker, pedigree, and environmental information could result in better prediction accuracy. When the interaction with environments is studied, the number of environments plays an important role because the number of correlations that needs to be estimated grows exponentially, and the complexity of the model increases significantly. The marker × pedigree interaction would indicate interactions between QTL and the genetic (pedigree) background, or simply a discrepancy between the pedigree data and the marker data. When the pedigree is deep, the expected additive genetic relationship should be close to the realized estimated relationship obtained by markers. In real plant breeding and animal data, most of the plant lines or animals evaluated in one year are not repeated the following year, and thus interaction terms between genetic (pedigree or marker) information and environments can only be assessed by the link between individuals established by pedigree and/or markers. Thus, it is possible to borrow information for predicting unobserved lines in new environments by using pedigree, marker, and correlated environmental information. However, it is acceptable that some degrees of confounded information’s between pedigree, genomic, and environments would exist in large plant breeding trials when not all lines are tested in all environments and the degree of overlapping of lines across years (or environments) is not high.

To answer some of the questions and considerations described above, our study aimed to assess the impact, over and above the main effects of pedigree and molecular markers, by including several types of interactions in prediction models. In particular, we contrasted models with interactions between pedigree and markers, and between pedigree, markers, and environments, with respect to models studied previously. Eight models that incorporated pedigree and dense molecular markers were tested. Our data included phenotypic information collected by the CIMMYT Global Wheat Program on first-year yield trial panels that evaluated 35,403 lines during 2013-2014 (13-14), 2014-2015 (14-15), 2015-2016 (15-16), and 2016-2017 (16-17), and none of the lines were tested in more than one year. Five traits were considered in the prediction study: grain yield (GY, ton ha^-1^), days to heading (DTH), days to maturity (DTM), plant height (PH, in cm), and lodging score (LD, 0-5). None of the wheat lines evaluated in one year were evaluated the following year; thus any attempt to study genetic (pedigree and markers) × environment interactions relies on the connectivity between lines across years based on pedigree and/or markers. The depth of the pedigree used in this study was 5 generations back, and most family sizes varied a great deal. The fact that none of the lines were repeated the following year does not prevent estimating the pedigree or genomic × environment interaction; the association between lines due to pedigree and/or genomic interaction establishes the link that makes it possible to estimate and account for these interactions.

## Materials and Methods

Phenotypic, pedigree, and molecular marker data for 35,403 wheat genotypes developed as described in Juliana *et al.* (2018a) were used in this study. Briefly, this information corresponds to four observation periods of the breeding pipeline: 7,577 genotypes for 13-14; 8,901 genotypes for 14-15; 9,311 genotypes for 15-16; and 9,614 genotypes for 16-17. Genotypes were derived from 5,369 crosses with family sizes ranging between 1 (1,017 families) and 116 (1 family). For example, there were 1,172, 301, 92, 32, and 10 families with at least 10, 20, 30, 40, and 50 individuals, respectively. The pedigree relationship between members of each family was identical, while marker-based relationships varied because of segregation effects due to recombination and Mendelian sampling. A total of 5,369 families were observed across periods; however, in some cases, individuals of these families were observed in 2 and 3 different periods (not the same genotypes but belonging to the same family). Out of the total number of families, 4,585 were observed exclusively in only one cycle (13-14; 14-15; 15-16, or 16-17); 775 in two cycles, and 9 in 3 cycles. In cycle 13-14, there were 1,382 families with sizes ranging from 1 to 51, in cycle 14-15 there were 1,294 families with sizes ranging from 1 to 48, in cycle 15-16 there were 1,332 families with sizes ranging from 1 to 65, and in cycle, 16-17 there were 2,154 families with sizes ranging from 1 to 116. It should be noted that the number of lines reported by Juliana *et al.* (2018a) corresponds to a subset of lines reported in this study; thus the number of wheat lines reported by Juliana *et al.* (2018a, b) is lower than the number of wheat lines reported in this study.

### Phenotypic data

Phenotypic data were recorded for the four aforementioned breeding periods/years (13-14, 14-15, 15-16, and 16-17). The traits that we considered were GY (ton ha^-1^), DTH, DTM, PH, and LD. The total number of wheat lines in each year were 7,577 (cycle13-14), 8,901 (cycle 14-15), 9,311 (cycle 15-16), and 9,614 (cycle 16-17). There were 200 to 350 hundred trails in each of the breeding periods. Each trial had 30 wheat lines organized in 6 incomplete blocks of size 5, and analyzed as incomplete block design with recovery of inter-block information, and incomplete block design plus spatial analyses using autoregressive in the row direction × autoregressive in the column direction model to account for soil variability. A final combined analysis was performed across all trails, and Best Linear Unbiased Estimate (BLUE) for all wheat lines was computed. All the 200-350 trails in each of the breeding periods were sown under full irrigation at the wheat experimental stations in Cd. Obregon, Sonora, México.

### Genotyping data

Genome-wide markers for the 46,089 lines were obtained as described by Juliana *et al.* (2018b). Initially, 11,293 markers were obtained and, after quality control, 6,978 SNPs remained for analysis. Markers with more than 30% missing values and a minor allele frequency smaller than 0.05 were discarded. After edits, we had pedigree, marker, and phenotypic information for 35,403 wheat lines and 6,978 SNPs.

### Statistical genome-enabled prediction models

The predictive ability of eight models was evaluated by assessing GY, DTH, DTM, PH, and LD as target traits. These models considered the main effects of pedigree and/or markers, and different types of interactions between pedigree, markers, and environments. The interactions were included by implementing the model of Jarquín *et al.* (2014).

All of the terms were entered as random effects into the model using co-variance structures. Variance components for each of the eight models were obtained under a Bayesian framework using the complete data set (*i.e.*, no missing values allowed). The eight models are described below.

### M1 - Environment + Pedigree model

This model is written as

$$y_{ij}=\mu +E_{i}+a_{j}+e_{ij},$$

where $y_{ij}$ denotes the phenotypic value for the *j*^th^ genotype tested in the *i*^th^ environment; *μ* is the overall mean, *E_i_* (*i* = 1,…, *I*) is a random environmental effect such that $E_{i}\overset{\text{iid}}{∼}N\left(0,\sigma_{\text{E}}^{2}\right)$ with $\sigma_{\text{E}}^{2}$ as the variance component; $a_{j\;}$(*j* = 1,…, *J*) is a random additive genetic effect for line *j* and ***a***={$a_{j}$} is assumed to follow the multivariate normal distribution *N*(**0**, $A\sigma_{\text{a}}^{2}$), where **A** is the numerator relationship matrix computed from pedigree information, and $\sigma_{\text{a}}^{2}$ is the additive genetic variance component; $e_{ij}$ is the random residual term with $e_{ij}\overset{\text{IID}}{∼}\text{N}\left(0,\sigma^{2}\right)$ and IID stands for independent and identically distributed. This model produces the same estimated breeding value for all individuals from the same family.

### M2 - Environment + Pedigree + Pedigree × Environment model

M2 extends M1 such that an interaction, based on pedigree, between genotypes and environments (G×E) is included in the model. The M2 model is written as

$$y_{ij}=\mu +E_{i}+a_{j}+aE_{ij}+e_{ij},$$

where *μ*, $a_{j}$, and $E_{i\;}$are defined as before, and $a{\text{E}}_{ij}$ is the interaction between the additive value of the *j*^th^ genotype and the *i*^th^ environment. As described by Jarquín *et al.* (2014), this interaction effect can be handled using covariance structures such that $\mathrm{aE}∼N\left(0,\left(Z_{g}AZ_{g}^{\text{'}}\right){}^{\circ}\left(Z_{E}Z_{E}^{\text{'}}\right)\sigma_{\text{aE}}^{2}\right)$, where $Z_{g}$ and $Z_{E}$ are incidence matrices for the lines and environments, respectively, ‘°’ denotes the Hadamard (element by element) product between two matrices, and $\sigma_{\text{aE}}^{2}$ is the corresponding variance component of the random interaction term ***aE***.

### M3 - Environment + Genome model

M3 is similar to M1 but instead of pedigree-based additive effects, it includes a main effect term for the genome-based (marker) breeding value. The model is

$$y_{ij}=\mu +E_{i}+g_{j}+e_{ij},$$

where *μ*, $E_{i}$, and $e_{ij}$ are as for M1. Here, $g_{j}\;$corresponds to the genomic breeding value of the *j*^th^ line defined as a linear combination of marker codes and the corresponding marker effects such that $g_{j}=\overset{p}{\underset{m=1}{\sum }}x_{jm}b_{m},\;$ where *p* is the number of markers, $x_{jm}$ is the marker code for the *j*^th^ line at the *m*^th^ marker position (*m* = 1,…, *p*), and $b_{m}$ is the corresponding marker effect. The marker effects are assumed normally distributed variables such that $b_{m}\overset{\text{IID}}{∼}N\left(0,\sigma_{\text{b}}^{2}\right),$ with $\sigma_{\text{b}}^{2}$ being the variance component of the marker effects. The covariance matrix of the vector of genomic values ***g***={$g_{j}$} can be written as $\text{Cov}\left(g\right)=G\sigma_{\text{g}}^{2}$, where **G** is the genomic relationship matrix computed as $G=\frac{XX^{\prime }}{p}$, ***X*** is the standardized genotype matrix (by columns), *p* is the number of markers, and $\sigma_{\text{g}}^{2}=p\times \sigma_{\text{b}}^{2}$ denotes the genomic variance component. Hence, $g=\left\{g_{j}\right\}∼N\left(0,G\sigma_{\text{g}}^{2}\right)$. Since molecular marker information varies across individuals (even within the same family), the estimated breeding values are unique for each genotype.

### M4 - Environment + Genome + Genome ×Environment model

M4 is the same as M2, but the G×E interaction is based on marker data instead of pedigree information. The model is written as:

$$y_{ij}=\mu +E_{i}+g_{j}+gE_{ij}+e_{ij},$$

where $gE_{ij}$ is the component that collectively represents interactions between molecular markers of the *j*^th^ line and the *i*^th^ environment. The distributional assumption for this term is similar to the interaction term in M2, but using the **G** matrix instead of the **A** matrix, such that $\mathrm{gE}=\left\{gE_{ij}\right\}∼\text{N}\left(0,\left(Z_{g}GZ_{g}^{\text{'}}\right){}^{\circ}\left(Z_{E}Z_{E}^{\text{'}}\right)\sigma_{\text{gE}}^{2}\right)$, and $\sigma_{\text{gE}}^{2}$ is the variance component of the random interaction component gE. As pointed out by Basnet *et al.* (2019), if observations are sorted by environment, then the variance-covariance matrix $\left(Z_{g}GZ_{g}^{\text{'}}\right){}^{\circ}\left(Z_{E}Z_{E}^{\text{'}}\right)$ is a block-diagonal matrix whose structure is similar to the Marker × Environment model of Lopez Cruz *et al.* (2015).

### M5 - Environment + Genome + Pedigree model

This model combines M1 and M3 since it contains both main effects (pedigree [$a_{\text{j}}$] and genomic information [$g_{j}$]); it is written as:

$$y_{ij}=\mu +E_{i}+g_{j}+a_{j}+e_{ij,}$$

where all of the terms were previously defined.

### M6 - Environment + Genome + Pedigree + Genome × Environment + Pedigree × Environment model

This model extends M5 by adding pedigree × environment and genome × environment interactions. M6 can also be viewed as a combination of M2 and M4, and the model is

$$y_{ij}=\mu +E_{i}+g_{j}+a_{j}+gE_{ij}+aE_{ij}+e_{ij},$$

where all model terms were previously defined.

### M7 - Environment + Genome + Pedigree + Genome × Pedigree model

By introducing pedigree × marker interaction effects via covariance structures, this model extends M5 as

$$y_{ij}=\mu +E_{i}+g_{j}+a_{j}+ga_{j}+e_{ij,}$$

where $ga_{j}$ is the interaction between the genomic and pedigree-based breeding values of the *j*^th^ genotype. The interaction component is distributed as

$ga=\left\{ga_{j}\right\}∼N\left(0,\left(Z_{g}GZ_{g}^{\text{'}}\right){}^{\circ}\left(Z_{g}AZ_{g}^{\text{'}}\right)\sigma_{\text{ga}}^{2}\right)$ with $\sigma_{\text{ga}}^{2}$ being the corresponding variance component.

### M8 - Environment + Genome + Pedigree + Genome × Pedigree + Genome × Pedigree × Environment model

This is the most parameterized model, where M7 is enriched by adding the interaction among pedigree, markers, and environment as:

$$y_{ij}=\mu +E_{i}+g_{j}+a_{j}+ga_{j}+gaE_{ij}+e_{ij},$$

where $gaE_{ij}$ is a three-way interaction term among the genomic breeding values and the additive value of the *j*^th^ genotype and the *i*^th^ environment. The interaction term is assumed to follow the distribution $\mathrm{gaE}=\left\{gaE_{ij}\right\}∼\text{N}\left(0,\left(Z_{\text{g}}GZ_{\text{g}}^{\prime }\right){}^{\circ}\left(Z_{\text{g}}AZ_{\text{g}}^{\prime }\right){}^{\circ}\left(Z_{\text{E}}Z_{\text{E}}^{\prime }\right)\sigma_{\text{gaE}}^{2}\right),$ where $\sigma_{\text{gaE}}^{2}$ is the corresponding variance component.

### Cross-validation and validation schemes

The performance of the models for predicting the five traits was evaluated using the weighted average Pearson’s correlation coefficient between observed and predicted values. For this, within each year/period, the Pearson’s correlation coefficient between predicted and observed values was computed first. Then, these values were weighted by the ratio between the sample size for each year divided for the total number of observations across years, and the resulting values were summed up. Thus, the periods with the largest number of observation are given more importance in the weighted average. One random cross-validation scheme (CV1) and a leave-one-out validation method (V00) were implemented, and predicted values *vs.* observed values were compared within and across time periods. These schemes mimic real plant breeding situations. CV1 refers to the scheme where the performance of 20% of the wheat lines was not observed in any of the years (environments) and the rest of the wheat lines (80%) were already observed in the same target environments. For this scheme, a fivefold random partitioning (80% of data used as the training set, and the remaining 20% used as the testing set) was employed. Four folds were used for training the models for predicting the remaining fold. This procedure was repeated over the five folds, and the predictions from the testing fold (5 in total) were joined in a single vector. Then, Pearson’s correlation between predicted and observed values within the same environment were computed. The partitioning was repeated 20 times at random.

The objective of V00 was to predict the performance of all new lines in a new year. The leave-one-year (environment) out scheme was implemented to define the training and testing sets; since random partition was not possible, it was ran only once. Also, since the lines were observed only once across all periods, no records on the same genotype in different environments were available. A more detailed description of the cross-validation techniques can be found in Jarquín *et al.* (2017).

### Software and Data availability

The Bayesian Generalized Linear Regression (BGLR) R-package (Pérez and de los Campos, 2014) was used for fitting the previously described GS models. Genomic and phenotypic data can be found in hdl.handle.net/11529/10548169. This link contains the genomic relationship matrix (G.RDA), the pedigree matrix (A.RDA), and the G×A matrix (GA.RDA). Entries in the columns and rows of the **A**, **G**, and **GA** matrices align with rows in the phenotypic data (Column 1; GID in Y.csv). In addition, the link hdl.handle.net/11529/10548169 contains Figures S1 and S2 in the Supplementary Material.

## Results

### Variance components from full data

Table 1 presents the estimated percentage of total variance accounted for by A, G, E, interaction, and the residual (Res) variance components for the eight different models (M1-M8) and the five traits. We focus on GY because it is the most important breeding trait and because the general results were similar to other traits. For GY, when comparing M1 and M3, the environmental component E explained around 45% of the variance in both cases, while A captured 38%, a much larger fraction than G (13.9%). When interactions between A or G and E were included through models M2 and M4, the environmental component explained around 40% in both models, while the interaction between pedigree and environments (AE) and between markers and environments (GE) captured 21.7% and 16.0% of the total variance, respectively. The relative amount of variability represented by the interaction components in both models was similar, but the difference in the amount of variance explained by main effects in these models was larger: 17.6% for A in M2 and only 6.7% for G in model M4. In model M2 (pedigree-based), the residual term (Res) represented 21.8%, while it represented 36.1% in M4 (genome based). In M5, where A and G were the main effects, the residual fraction was the same as in M1. Here, the pedigree term (A) captured 36.4% of the phenotypic variability, while the markers (G) captured only 8.4%. In M6, the relative amounts of phenotypic variability explained by the variance components were: E (35.3%), A (12.3%), G (6.6), AE (20.3), and GE (7.1); the remaining 18.5% was accounted for by the residual term. Also in M6, we observed a similar picture as in M2 and M4: the AG and GE components took away a large amount of the phenotypic variability explained by main effects. This was not surprising, as the E term usually explains a large amount of phenotypic variability in multi-environment trials where interaction terms carry sizable information.

**Table 1:** Estimated percentage of the total variance accounted for by each random effect of the corresponding model for grain yield (GY), days to heading (DTH), plant height (PH), lodging (LD), and days to maturity (DTM)

		Estimated percentage of total variance explained by each component							
	Models	E	A	G	AE	GE	GA	GAE	Res.
**GY**	M1: E+A	44.5	37.6						17.9
	M2: E+A+AE	39.0	17.6		21.7				21.8
	M3: E+G	45.4		13.9					40.7
	M4: E+G+GE	41.3		6.7		16.0			36.1
	M5: E+G+A	37.6	36.4	8.4					17.6
	M6: E+G+A+GE+AE	35.3	12.3	6.6	20.3	7.1			18.5
	M7: E+G+A+GA	38.1	26.6	7.6			19.2		8.5
	M8: E+G+A+GA+GAE	29.9	41.8	5.7			5.7	10.9	6.0
									
		**Estimated percentage of total variance explained by each component**							
	**Models**	E	A	G	AE	GE	GA	GAE	Res.
**Heading**	M1: E+A	44.3	36.1						18.9
	M2: E+A+AE	36.8	19.4		21.7				22.1
	M3: E+G	43.9		19.5					36.6
	M4: E+G+GE	34.7		14.1		16.2			35.0
	M5: E+G+A	35.5	36.4	14.5					16.1
	M6: E+G+A+GE+AE	32.9	12.3	12.6	15.7	7.7			15.7
	M7: E+G+A+GA	42.4	26.6	11.9			21.9		4.8
	M8: E+G+A+GA+GAE	38.1	41.8	13.3			10.6	14.5	4.5
									
		**Estimated percentage of total variance explained by each component**							
	**Models**	E	A	G	AE	GE	GA	GAE	Res.
**Height**	M1: E+A	34.8	35.3						29.9
	M2: E+A+AE	24.5	22.9		18.5				34.1
	M3: E+G	34.3		17.6					48.1
	M4: E+G+GE	25.6		11.4		17.4			45.6
	M5: E+G+A	27.5	31.4	12.7					28.4
	M6: E+G+A+GE+AE	38.6	16.5	14.4	19.9	10.6			38.6
	M7: E+G+A+GA	24.6	21.7	12.1			23.5		18.1
	M8: E+G+A+GA+GAE	21.9	20.1	13.5			10.4	16.8	17.3
									
		**Estimated percentage of total variance explained by each component**							
	**Models**	E	A	G	AE	GE	GA	GAE	Res.
**Lodging**	M1: E+A	41.8	40.6						17.6
	M2: E+A+AE	38.2	23.4		18.5				19.9
	M3: E+G	43.7		15.8					40.5
	M4: E+G+GE	39.4		8.0		16.6			36.0
	M5: E+G+A	35.8	37.1	9.4					17.7
	M6: E+G+A+GE+AE	30.3	15.8	7.5	18.5	8.4			19.4
	M7: E+G+A+GA	36.9	22.8	8.5			23.8		8.0
	M8: E+G+A+GA+GAE	35.6	19.5	9.6			10.9	15.0	9.2
									
		**Estimated percentage of total variance explained by each component**							
	**Models**	E	A	G	AE	GE	GA	GAE	Res.
**Maturity**	M1: E+A	45.1	35.5						19.4
	M2: E+A+AE	43.2	15.6		19.8				21.4
	M3: E+G	48.0		16.6					35.5
	M4: E+G+GE	40.1		11.4		15.4			33.1
	M5: E+G+A	37.8	32.2	12.6					17.3
	M6: E+G+A+GE+AE	36.4	9.0	10.7	20.6	7.4			15.9
	M7: E+G+A+GA	43.4	20.0	10.8			20.2		5.6
	M8: E+G+A+GA+GAE	39.1	19.1	12.4			7.7	16.3	5.3

When the interaction between pedigree and markers (GA) was introduced using M7, the fraction of total variability explained by the different components was: E (38.1%), A (26.6%), G (7.6%), GA (19.2), and the residual term (8.5%). Including the GAE interaction in M8 reduced the fraction of residual variance the most (6.0%) compared to M1-M7, where the residual term captured between 40.7% and 8.5% of the phenotypic variability. In M8, the proportion of total variance was as follows: E (29.9%), G (5.7%), A (41.8%), GA (5.7%), and GAE (10.9%). It is clear that when interactions GA and GAE are included in the model, the fractions of variance accounted for by the residual term dropped remarkably in M7 and M8 compared to models that exclude GA. Results for other traits showed similar patterns, as already mentioned.

### Prediction: Random cross-validation CV1

Table 2 and Figure S1 (Supplementary Material, in hdl.handle.net/11529/10548169) display the results for each environment (mean and standard deviation across the 20 fivefold random partitions of the CV1 scheme) for grain yield (GY), days to heading (DTH), days to maturity (DTM), plant height (PH), and lodging (LD). For GY the weighted average Pearson’s correlation over the four years using M1 (*i.e.*, environment + pedigree, E+A) was 0.562. When the interaction between pedigree and environment (AE) was included via M2, this value increased slightly to 0.577 for GY. On the other hand, when only the main effect of markers (G) was included using M3 for GY, the mean correlation decreased to 0.458, but when GE was added via M4, it improved to 0.515.

**Table 2:** Weighted average (WA) Pearson’s correlation and standard deviation (SD) over 20 replicates of a fivefold (CV1) random cross-validation (prediction of new lines not observed in any year) for eight models (M1: environment + pedigree; M2: environment + pedigree + pedigree × environment; M3: environment + marker; M4: environment + marker + marker × environment; M5: environment + pedigree + marker; M6: environment + pedigree + marker + pedigree × environment + marker × environment; M7: environment + pedigree + marker + pedigree × marker; M8: environment + pedigree + marker + pedigree × marker + pedigree × marker × environment) for grain yield (GY), days to heading (DTH), days to maturity (DTM), plant height (PH), and lodging (LD) for a wheat breeding pipeline comprised of 35,403 lines observed in four periods/years (13-14, 14-15, 15-16 and 16-17). Lines were observed only once across all periods

Trait	Year	M1		M2		M3		M4		M5		M6		M7		M8	
		Mean	SD	Mean	SD	Mean	SD	Mean	SD	Mean	SD	Mean	SD	Mean	SD	Mean	SD
**Yield**	13-14	0.645	0.003	0.647	0.004	0.523	0.002	0.580	0.001	0.685	0.003	0.694	0.002	0.704	0.002	0.706	0.002
	14-15	0.482	0.002	0.492	0.002	0.411	0.003	0.483	0.005	0.540	0.002	0.560	0.002	0.561	0.003	0.570	0.004
	15-16	0.590	0.002	0.612	0.004	0.479	0.002	0.528	0.002	0.634	0.005	0.653	0.002	0.645	0.002	0.657	0.002
	16-17	0.544	0.003	0.565	0.002	0.430	0.001	0.482	0.003	0.577	0.002	0.597	0.002	0.590	0.003	0.599	0.004
	**W. Mean**	**0.562**	**0.003**	**0.577**	**0.003**	**0.458**	**0.002**	**0.515**	**0.003**	**0.606**	**0.003**	**0.623**	**0.002**	**0.622**	**0.002**	**0.630**	**0.003**
																	
**Heading**	13-14	0.592	0.004	0.594	0.004	0.564	0.001	0.610	0.001	0.666	0.003	0.675	0.004	0.690	0.004	0.696	0.004
	14-15	0.527	0.003	0.533	0.003	0.482	0.003	0.540	0.003	0.619	0.003	0.634	0.001	0.656	0.003	0.663	0.002
	15-16	0.537	0.003	0.570	0.003	0.537	0.001	0.588	0.003	0.636	0.001	0.667	0.002	0.655	0.001	0.668	0.002
	16-17	0.546	0.003	0.563	0.004	0.549	0.001	0.583	0.002	0.648	0.003	0.665	0.003	0.670	0.005	0.679	0.004
	**W. Mean**	**0.549**	**0.003**	**0.564**	**0.004**	**0.532**	**0.001**	**0.579**	**0.002**	**0.641**	**0.003**	**0.660**	**0.002**	**0.667**	**0.003**	**0.675**	**0.003**
																	
**Maturity**	13-14	0.588	0.005	0.590	0.003	0.543	0.001	0.590	0.003	0.666	0.004	0.675	0.004	0.689	0.003	0.696	0.009
	14-15	0.523	0.004	0.528	0.003	0.496	0.002	0.552	0.002	0.623	0.003	0.635	0.003	0.651	0.002	0.656	0.004
	15-16	0.597	0.003	0.631	0.002	0.534	0.002	0.578	0.003	0.663	0.002	0.697	0.001	0.676	0.001	0.696	0.003
	16-17	0.535	0.002	0.567	0.002	0.541	0.002	0.583	0.002	0.628	0.001	0.658	0.002	0.648	0.003	0.666	0.006
	**W. Mean**	**0.560**	**0.003**	**0.579**	**0.003**	**0.528**	**0.002**	**0.575**	**0.003**	**0.644**	**0.002**	**0.666**	**0.002**	**0.665**	**0.002**	**0.678**	**0.005**
																	
**Height**	13-14	0.520	0.004	0.524	0.004	0.440	0.004	0.483	0.003	0.567	0.001	0.577	0.002	0.589	0.002	0.596	0.004
	14-15	0.462	0.001	0.466	0.002	0.434	0.002	0.476	0.003	0.518	0.003	0.526	0.003	0.533	0.002	0.539	0.004
	15-16	0.525	0.002	0.549	0.001	0.459	0.003	0.502	0.004	0.583	0.002	0.604	0.003	0.597	0.002	0.610	0.002
	16-17	0.513	0.002	0.538	0.003	0.441	0.001	0.518	0.001	0.567	0.001	0.596	0.001	0.581	0.002	0.593	0.003
	**W. Mean**	**0.505**	**0.002**	**0.520**	**0.002**	**0.444**	**0.002**	**0.496**	**0.003**	**0.559**	**0.002**	**0.577**	**0.002**	**0.575**	**0.002**	**0.585**	**0.003**
																	
**Lodging**	13-14	0.562	0.005	0.564	0.006	0.499	0.004	0.541	0.005	0.616	0.003	0.624	0.005	0.630	0.005	0.631	0.005
	14-15	0.503	0.006	0.507	0.006	0.464	0.001	0.480	0.002	0.568	0.005	0.575	0.005	0.586	0.005	0.592	0.005
	15-16	0.559	0.005	0.579	0.004	0.358	0.003	0.422	0.006	0.566	0.002	0.595	0.003	0.580	0.004	0.595	0.004
	16-17																
	**W. Mean**	**0.541**	**0.005**	**0.550**	**0.005**	**0.436**	**0.002**	**0.477**	**0.004**	**0.582**	**0.003**	**0.597**	**0.004**	**0.597**	**0.005**	**0.605**	**0.004**

When combining the main effects of environments, markers and pedigree (E+A+G) using M5, for GY the correlation increased to 0.606. Even better results were obtained for GY when both AE and GE were added to the previous model using M6. In this case, the predictive correlation increased to 0.623, so these results were better than when using models M1-M5 for all years/periods for GY. M7 combining A, G and G×A gave a Pearson’s correlation of 0.622. M7 produced better results than M1-M5 for GY for all periods/years and better results than M6 for two out of the four years. M8, an extension of M7 that also considers G×A×E interaction, increased the correlation to 0.630. M8 was the best model for all years.

The most effective model (M8) improved predictive ability for GY by 12.1% and 37.5% over models that included only main effects of pedigree and markers (M1 and M3). The year when prediction accuracy was best was 13-14 for all models, and the year with the worst prediction outcome was 14-15. Days to heading (DTH) showed patterns that were similar to those of GY. The weighted average correlations ranged between 0.532 and 0.675, with M3 and M8 delivering the lowest and highest values, respectively. For DTH, M8 performed better than the other models (M1-M7) in all years. For days to maturity (DTM), the weighted average Pearson’s correlation for all models ranged between 0.528 and 0.678, and as they did for the other traits, M3 and M8 delivered the worst and best results, respectively. The average Pearson’s correlation for plant height (PH) ranged between 0.444 and 0.585. Again, M3 and M8 produced the worst and best results, respectively. The mean Pearson’s correlation for Lodging ranged between 0.436 (M3) and 0.605 (M8).

### Prediction: Leave-one-year-out validation (V00)

Results for grain yield (GY), days to heading (DTH), days to maturity (DTM), plant height (PH), and lodging (LD) obtained with the V00 scheme are provided in Table 3 and Figure S2 (Supplementary Material in hdl.handle.net/11529/10548169). For all traits, the model with a main effect of markers (M3) outperformed the other seven models most of the time for all periods/years. There were exceptions for plant height, where M5-M8 performed equally or slightly better than M3. For V00, none of the models including interactions provided better average correlations than M3, as occurred with the CV1 scheme, where accommodating interactions increased the prediction accuracy obtained by models with main effects only. For yield (GY), the mean Pearson’s correlation over all models ranged between 0.072 (M2) and 0.293 (M3). When main effects of markers and pedigree were combined in M5, the predictive ability decreased to 0.250. Pearson’s correlations for the best model (M3) for periods 13-14, 14-15, 15-16, and 16-17 were 0.336, 0.264, 0.350, and 0.231, respectively.

**Table 3:** Weighted average (WA) Pearson’s correlation for validation V00 (prediction of new lines observed only in one year - leaving one year out at a time as testing set) for eight models (M1: environment + pedigree; M2: environment + pedigree + pedigree × environment; M3: environment + marker; M4: environment + marker + marker × environment; M5: environment + pedigree + marker; M6: environment + pedigree + marker + pedigree × environment + marker × environment; M7: environment + pedigree + marker + pedigree × marker; M8: environment + pedigree + marker + pedigree × marker + pedigree × marker × environment) for grain yield (GY), days to heading (DTH), days to maturity (DTM), plant height (PH), and lodging (LD) for a wheat breeding pipeline comprised of 35,403 lines observed in four periods/years (13-14, 14-15, 15-16 and 16-17). Lines were observed only once across all periods

Trait	Year	M1	M2	M3	M4	M5	M6	M7	M8
**Yield**	13-14	0.125	0.053	0.336	0.141	0.314	0.261	0.305	0.313
	14-15	0.084	0.016	0.264	0.231	0.202	0.154	0.182	0.226
	15-16	0.202	0.185	0.350	0.219	0.316	0.284	0.295	0.283
	16-17	0.113	0.030	0.231	0.127	0.181	0.122	0.212	0.147
	**W. Mean**	**0.131**	**0.072**	**0.293**	**0.181**	**0.250**	**0.203**	**0.246**	**0.238**
									
**Heading**	13-14	0.060	0.083	0.325	0.244	0.283	0.195	0.266	0.298
	14-15	0.115	0.087	0.281	0.197	0.267	0.245	0.267	0.261
	15-16	0.250	0.190	0.409	0.393	0.369	0.312	0.384	0.359
	16-17	0.069	0.096	0.309	0.146	0.277	0.219	0.255	0.247
	**W. Mean**	**0.126**	**0.116**	**0.332**	**0.245**	**0.300**	**0.245**	**0.294**	**0.291**
									
**Maturity**	13-14	0.072	0.055	0.321	0.270	0.237	0.308	0.263	0.253
	14-15	0.122	0.076	0.309	0.270	0.278	0.308	0.293	0.272
	15-16	0.221	0.266	0.416	0.329	0.365	0.260	0.369	0.328
	16-17	0.133	0.125	0.338	0.273	0.319	0.306	0.292	0.270
	**W. Mean**	**0.140**	**0.135**	**0.347**	**0.286**	**0.304**	**0.295**	**0.306**	**0.282**
									
**Height**	13-14	0.146	0.124	0.314	0.275	0.314	0.212	0.305	0.279
	14-15	0.111	0.107	0.293	0.259	0.250	0.251	0.250	0.223
	15-16	0.216	0.223	0.362	0.187	0.321	0.291	0.319	0.366
	16-17	0.130	0.100	0.217	0.196	0.243	0.258	0.240	0.243
	**W. Mean**	**0.151**	**0.139**	**0.295**	**0.226**	**0.281**	**0.255**	**0.277**	**0.278**
									
**Lodging**	13-14	0.200	0.175	0.403	0.315	0.340	0.221	0.338	0.296
	14-15	0.133	0.071	0.248	0.168	0.198	0.115	0.193	0.211
	15-16	0.136	0.096	0.233	0.229	0.228	0.147	0.208	0.187
	16-17								
	**W. Mean**	**0.154**	**0.110**	**0.288**	**0.233**	**0.251**	**0.158**	**0.241**	**0.227**

The average Pearson’s correlation for days to heading (DTH) ranged between 0.116 (M2) and 0.332 (M3). The model with A and G produced a mean accuracy of 0.3. For the four periods/years, Pearson’s correlations obtained with M3 were 0.325, 0.281, 0.409, and 0.309. Results for days to maturity (DTM) produced a mean Pearson’s correlation of 0.135 for the worst model (M2), and 0.347 for the best model (M3). Pearson’s correlations obtained with M3 were 0.321, 0.309, 0.416, and 0.338 for periods 13-14, 14-15, 15-16, and 16-17, respectively.

For plant height (PH), the average Pearson’s correlations for the worst and the best models were 0.139 (M1) and 0.295 (M3). Although M3 produced the best results on average, in some years, M5-M8 performed the same (M5) or slightly better (M6-M8) than M3. Results for Lodging were in line with results for the other traits. The mean Pearson’s correlation varied between 0.110 (M2) and 0.288 (M3). For the best model, correlations for the three years 13-14, 14-15, and 15-16 were 0.403, 0.248, and 0.233, respectively.

## Discussion

Genome-enabled prediction is a tool for plant breeders that has the potential to increase genetic gain for high yielding wheat varieties with agronomically desired traits. Since one of the main objectives of wheat breeding is to develop varieties that are insensitive to environmental changes, it is important to develop prediction models that incorporate information not only on dense molecular markers and pedigree, but also interactions with the environment. We implemented models that use the reaction norm concept and incorporate the interactions among the terms via a covariance (kernel) structure, because this approach enables the inclusion of high dimensional data. We evaluated eight models for predicting five traits using a cross-validation (CV1) and a leave-one-year-out validation scheme (V00).

### Cross-validation prediction accuracy

The trend was similar for predicting all traits in terms of model predictive ability under both validation schemes (CV1 and V00). However, the two validation schemes showed differences. For CV1, accounting for the environment via interactions with genomic sources seemed more beneficial than for V00. Perhaps this is due to the fact that CV1 allows borrowing information from other environments to increase predictive ability, whereas in V00 we predicted the performance of unobserved lines in unobserved environments, certainly a very complex task, specially under unpredictable year conditions. One of the main goals of this study was to examine whether including the interaction between marker and pedigree information would enhance prediction accuracy. When we predicted the performance of unobserved lines in unobserved environments (V00), there was no advantage in including GA interaction. However, under CV1, M8 outperformed all models for most traits and periods (years). In addition to the main effects of E, A, and G, M8 included GA and GAE interactions.

A main observation in our study was that including pedigree information in the model instead of G was better for within-year CVs, whereas markers had advantages across years. This is consistent with observations of elite yield trials made by Juliana *et al.* (2018 a, b). These results utilizing various types of information may enhance prediction problems in the breeding pipeline, as mentioned by Morota and Gianola (2014) and Gianola *et al.* (2014) when discussing multi-kernel approaches to prediction. Another possible reason why pedigree-based models (M1 and M2) outperformed marker-based models (M3 and M4) for the CV1 scheme is that some families (∼15%) overlapped, meaning that they were observed in more than one year, enabling to borrow information across periods.

### Pedigree × genomic and pedigree × genomic × environment interactions

It has been argued that genome-enabled prediction should work well when there is genetic relatedness among lines (as indicated by the pedigree), but not otherwise. In other words, the importance of a genome-based model depends on the strength of pedigree relationships (Habier *et al.*, 2007). In a model with pedigree, genomic information, and their interaction, as in model M7 (A+G+GA), the expected phenotypic change per unit of genomic relatedness depends on the level of genetic relationship conveyed by the pedigree. We found that the amount of variance explained by GA in model M7 was between 19.2% and 23.8%, a sizable amount.

However, the phenotypic change per unit of genomic relatedness may be influenced not only by pedigree relationships, but also by variation in environment. This three-factor interaction (GAE) explained a sizable proportion of the within environment variance for the different traits. For grain yield, it explained 10.9% of the variability in model M8 (A+G+GA+GAE), and was also the genome-based prediction model for predicting all periods (13-14, 14-15, 15-16, and 16-17) under CV1. Under V00, the impact of the interactions was negligible and the main determining factor was G (marker); thus including the genome-based information produced the best prediction accuracy for all models in the environments.

A wide range of variation in genomic relatedness in a given genetic relationship is expected with large family sizes. In this data set, the family size varies; as already mentioned, family sizes range between 1 (1,017 families) and 116 (1 family). There were 1,172, 301, 92, 32, and 10 families with at least 10, 20, 30, 40, and 50 individuals, respectively. Perhaps with more families with larger family sizes, the effect of variation in the genomic relationship per unit of genetic relatedness (pedigree) could increase, in which case GA and GAE interactions could influence prediction accuracy more strongly. The complex three-factor interaction, GAE, may also capture cryptic, small, epistatic effects, which would change from environment to environment and should be accounted for by the GAE term.

Interestingly, Morota *et al.* (2014) observed that when kernels are mutually orthogonal to each other could enhance predictive ability; however, these are not easy to construct. On the other hand, when these are not mutually orthogonal the partition of variance is difficult to interpret mechanistically. Hence, the partitioning of variance into A, G, and GA may reflect “collinearity” among kernels. Therefore, while a multi-kernel approach may be more effective for prediction, the interpretation of variance components should be interpreted with caution.

### What variability is accounted for pedigree, genomic and their interaction?

It is expected for **A**◦**G** to be similar to the **G**◦**G** matrix, which has been used to model pairwise additive × additive epistasis (Jiang and Reif, 2015). These models have been shown to be effective in increasing genomic prediction accuracy (Crossa *et al.,* 2010 and Martini *et al.,* 2016 using the 599 CIMMYT wheat lines). It is possible that in this study **A** and **G** would be dissimilar enough to produce a covariance structure that would differ from the expected additive × additive epistasis covariance. Therefore, the covariance **A**◦**G** may simply be picking up epistasis. Biologically it is not easy to explain exactly what variation A◦G captures compared to A◦A and G◦G, but mathematically it explains the interaction among loci (epistasis) that is accounted by the interaction of the marker and pedigree information (instead of accounting for only the marker relatedness). The more complex model, M8, would therefore simply include the epistasis term by environment interaction.

In general, pedigree shrinks the additive covariance within family to 0, emphasizes closer relationships and deemphasizes further relationships (*i.e.*, “unrelated” lines do share some common ancestor, and is dependent on pedigree depth). Markers may produce unbiased estimates depending on their distribution in the genome, and the filtering methods used (*e.g.*, redundant markers are often discarded). Therefore, it can be speculated that **G**◦**G** (and similarly **A**◦**G**) would also tend to overemphasize close relationships while deemphasizing further relationships.

Arguably, for a G+A model, matrix A picks up some genetic variance that is not tagged by G (markers). In livestock, markers mainly pick up realized relatedness (better than A) as opposed to QTL effects. On the other hand, in humans with high density SNP markers and weak pedigree relationships, the markers would pick up mainly QTL effects. In wheat, it is possible that a G constructed with high density markers would pick up a combination of line, pedigree, and QTL effects. Unfortunately, since A and G kernels are not mutually orthogonal, the interpretation of variance component estimations is ambiguous.

Several studies indicated that genome-enabled prediction works when training and testing sets are genetically related, but that it does not work when genetic relationships are weak (where the definition of weak and strong relatedness highly depends on the species in question, but mathematically speaking the relatedness can be captured by the correlation of the genetic marker or pedigree information corresponding to the individuals in the testing/training sets). This phenomenon could be interpreted as one in which there is interaction between A and G. In other words, if A encodes strong genetic relatedness, a G model may be useful. Conversely, if A is sparse, a G model may not help prediction. This basic idea could be exploited predictively in a model with A+G+GA kernels. We hypothesized that the GA interaction kernel would capture such a phenomenon within a population and evaluated the conjecture with real data.

### Conclusion

GS is an important part of a breeding program’s selection and decision making. Increased prediction accuracy can reduce the cost of a breeding pipeline, increase the selection intensity and shorten the selection cycles, thus increasing the genetic gain per cycle. Since the environmental factors are a big part of how a variety performs, it is also important to consider environmental information when building prediction models for GS. It has also been shown that GS models can benefit from the inclusion of pedigree information. Thus, in this study we compared models including marker genotype, pedigree, and environmental information in various combinations. Some of the models included main effects only, and some included interactions. We found that, depending on the validation scheme, including the interaction between the marker genotypes, pedigree, and environmental factors can be beneficial, highlighting the importance of developing future GS models where other sources of information and their interactions are considered, with the potential to increase prediction accuracy.
